# “Don’t Study Us Without Us”: Age Inclusivity in Research

**DOI:** 10.1093/geroni/igaf122.135

**Published:** 2025-12-31

**Authors:** Nina Silverstein, Lauren Bowen, Joann Montepare

## Abstract

The Research Domain of the Age Inclusivity Domains in Higher Education (AIDHE) model endorsed by GSA advocates for facilitating aging-related research by faculty and students with attention to age-diversity, intergenerational dynamics, and interdisciplinary collaboration; incorporating age-inclusive perspectives into research agendas and scholarly activities across disciplines; and expanding knowledge to inform applications and interventions. Whitbourne and colleagues’ (2024) study that surveyed 6,831 faculty, staff, and students across 25 U.S. institutions revealed that only about 61% of age-inclusive research practices were in place across campuses. This symposium will show creative ways these practices can be expanded in higher education. Whitbourne will begin by describing the AIDHE model and what their study revealed about the prevalence and range of age-inclusive research practices. McGaffigan will present examples from the Aging PCOR Learning Collaborative to show how involving older adults as experts fosters meaningful research and outcomes. Waterhouse adds the perspective of a medical campus and will describe a workforce development program for older adults to become research specialists and be eligible for hire onto research teams; and their Research Roadshows and Living Labs that provide experiential opportunities for older adults to engage directly with research teams. Finally, Koren will consider the value of age-diversity in educational research that involves older learners, younger learners, and faculty to provide insights about the effectiveness of intergenerational classrooms. Montepare will serve as discussant and reflect on strategies for strengthening age-inclusive practices in this critical domain.

